# Aftermath of the COVID-19 Pandemic: Resilience and Mental Health of Emerging Adult University Students

**DOI:** 10.3390/ijerph20206911

**Published:** 2023-10-11

**Authors:** Sophie Leontopoulou

**Affiliations:** Department of Primary Education, University of Ioannina, 45110 Ioannina, Greece; sleon@uoi.gr

**Keywords:** COVID-19 impacts, resilience, mental health, moderation, emerging adults

## Abstract

This study explored the relationship between COVID-19 impacts and resilience in emerging adulthood during the final two months of the pandemic. It aimed to examine whether mental health symptoms moderated this relationship. In total, 205 university students completed an online questionnaire survey. Regression analysis was used to examine the prediction of resilience by pandemic-related impacts, and moderation analysis was used to explore the potential moderating effect of mental health on the relationship between impacts and resilience. The findings failed to confirm the hypothesis that total COVID-19 impacts would predict resilience. Rather, resource-type impacts predicted resilience [*B* = 0.17, *p* < 0.5]. Significant partial correlations found among resource, financial, and psychological impacts may go some way toward clarifying connections between impacts and resilience [for resource-type impact with financial-type impact, *r* = 0.48, *p* < 0.01; for resource-type impact with psychological impact, *r* = 0.22, *p* < 0.01]. The results confirmed the hypothesis that mental health symptoms would moderate the relationship between pandemic impacts and resilience [for the overall model, *R* = 0.41, Δ*R*^2^ = 0.16, *MSE* = 0.76, *F* (4, 200) = 10.19, *p* < 0.001; for the interaction between total COVID impacts and resilience, Δ*R*^2^ = 0.017, *F* (1, 200) = 3.98, *p* < 0.05]. Thus, emerging adult students with low or moderate levels of symptomatology were more resilient, independently of the level of pandemic-related stressors they faced. Those experiencing higher levels of mental health symptoms, in tandem with high levels of pandemic-related impacts, exhibited increasingly higher resilience levels [*b* = 0.17, 95% CI [0.02, 0.32], *t* = 2.26, *p* = 0.025]. These youths may be better equipped to handle severe stress and adversity thanks to skills and resources they possess and are experienced in using. The implications of these findings for each group of young people are discussed for their usefulness in directing future research and interventions to foster resilience during current and future crises and health pandemics.

## 1. Introduction

The World Health Organization declared the COVID-19 outbreak a pandemic in March 2020; it declared an end to COVID-19 as a public health emergency in May 2023, albeit stressing that the disease still remained a global threat [1]. By that time, more than 765,000,000 people were diagnosed with the disease and over 7 million people lost their lives to it. Moreover, over 100 countries worldwide took drastic measures to contain the spread of the disease, including lockdowns and restrictions on social activities and contacts as well as in trade, worship, education, and leisure activities. This enforced severe deviation from everyday conduct and routines proved to have negatively affected most aspects of life, including mental health, well-being, and resilience in all age groups across the globe. As far as emerging adults are concerned, a newly established age group spanning the ages between 18 and 29 years [2], UNESCO [3] reported that the pandemic impacted over 1.5 million youths and students and that the most vulnerable learners were hit the hardest. The closure of schools and universities during the lockdowns and the enforced overnight migration from face-to-face to online teaching modes seem to have affected the mental health and well-being of studying youths [4]. This paper aims to explore the impacts of COVID-19 on the resilience and mental health of emerging adult university students in the aftermath of the pandemic. In so doing, it hopes to clarify the relationships between the variables, to draw pathways to resilient adaptation, and ultimately to provide directions toward enhancing positive youth development under conditions of crisis. 

Mental health and its many aspects, including emotional, social, financial, and physical health, are currently at the forefront of public health concerns worldwide. The study of the impact of COVID-19 on mental health, well-being, and resilience, especially during the various lockdown and containment measures imposed around the globe are identified as research priorities [5,6]. A number of studies since the beginning of the pandemic attempted to explore different aspects of mental health and well-being in diverse populations. Fewer studies directly assessed the impact of pandemic-related phenomena on resilience or indeed the relations between resilience and mental health in the light of the COVID-19 pandemic. Moreover, there needs to be more understanding of the nature of such relations during emerging adulthood, an important time in the lives of young people, including those studying toward a degree. During the lockdowns, the quality and nature of education and training can be affected to a larger or lesser degree, thus negatively impacting the future prospects of youths in terms of finding suitable employment, alongside their psychosocial well-being [7,8,9,10]. The present study explores how studying youths achieve resilient outcomes given their mental health status as affected by pandemic restrictions in various aspects of their lives. The following sections depict key issues in the above theme. 

The next subsection deals with issues around resilience and its relationship to mental health during crises. It focuses on the latest health crisis, namely the COVID-19 pandemic and its impacts on resilience and mental health.

### 1.1. Resilience and Mental Health in the COVID-19 Era

Resilience is a dynamic process of adaptation despite risks and challenges [11]. Since its inception resilience has been studied within a framework including risk and adversity as well as crises and disaster [12]. Crises such as health, socio-economic, environmental, or other crises represent adverse life conditions that challenge the adaptation system of individuals. The COVID-19 pandemic represents the latest and most severe crisis that people around the globe had to face. Since the outbreak of this latest pandemic, many research efforts concentrated on understanding its impact on resilience and the mental health of different population groups around the globe have been undertaken. It is generally understood that, during the pandemic, the prevalence of mental health problems, including stress, anxiety, and depression in the general population, was around 30% [13,14,15]. Resilience is consistently reported to be inversely related to mental health problems, such as distress, loneliness, anxiety, depression, and suicidal ideation [16], especially during natural disasters [17]. 

Masten and Motti-Stefanidi [18] suggested that COVID-19 represents a multisystem crisis and disaster that affects individual, family, organizational, and community resilience at many levels. The authors proposed a number of psychosocial factors that promote multisystem resilience during disasters. At the individual level for children and youths, these included close relationships, agency, active coping, problem solving, hope, meaning and purpose, positive routines, etc. Indeed, during the latest pandemic, resilience was reported to be lower than published norms [15]; still, it is believed to relate to modifiable factors during the lockdowns, such as social support factors and daily activities. Recently, other authors also attributed a protective role to resilience, either a mediating or a moderating one, with respect to adverse effects of the pandemic [9,19,20]. Resilience is alternatively considered and measured as a well-being outcome to adverse situations, to be assessed in its own right using various resilience scales or to be inferred by the use of proxy variables, ranging from self-control and respect for parents to internet- and mobile-phone-accessed information about epidemics and other scenarios. [11,12,13,14,15,16,17,18,19,20,21,22,23]. Independently of differences in its conceptualization, resilience is invariably thought to play a key role in positive adaptation throughout the life cycle. In the present study, resilience is examined as a well-being indicator under the influence of COVID-19-related adverse impacts in emerging adulthood. 

The following subsection examines the interplay of resilience and mental health under pandemic-imposed conditions for emerging adult students. Psychosocial characteristics of the latter interact with external conditions, such as the COVID-19 pandemic, and may lead to differential developmental trajectories, as indicated below. 

### 1.2. Exploring Resilience and Mental Health of Emerging Adult Students during the Pandemic

Emerging adults, as proposed by Arnett [2], actively seek independence from parents, and dynamically explore possibilities in terms of work, study, and financial self-sufficiency, but also romantic relations, friendships, and socialization at large, as well as world views and ideology. The inherent instability of this age group, as described by Arnett [2], alongside pervasive feelings of being ‘in-between’ adolescence and adulthood, may lead to disruptions in their developmental trajectories, especially in times of crisis. It follows that the additional disruptions brought about by the COVID-19 pandemic at various levels (including, but not limited to, physical and mental health care, personal and social life, education and work), may affect emerging adults disproportionately and impact the mental health of young people negatively [24]. Holmes and colleagues [5] reported that in the UK, after the first months of the pandemic and lockdown measures, young adults, and especially young men, were more negatively affected in terms of psychological distress (by 0.50 standard deviations). In a longitudinal UK study, Stroud and Gutman [25] identified risk factors for poorer mental health throughout the pandemic. These included being female, having a lower income, as well as a pre-existing mental health condition. Alonzi and colleagues [26] identified groups of young adults who are at increased risk for developing mental health problems during the pandemic. In particular, with respect to gender, non-binary participants, followed by females, were more likely to report depression and anxiety. Young people with both mental and physical health problems also reported the highest levels of anxiety and depression, followed by those with mental health problems alone. 

Some researchers are concerned that emerging adult students are even more prone to developing mental health problems, such as anxiety and depression, during the COVID-19 pandemic [24,27], since disruptions in education, work, and social life may adversely affect their economic and career prospects as well as social contacts [26]. Recent studies reported that almost a quarter of Chinese college and university students exhibited anxiety as a result of the recent pandemic [28]. In a Greek study carried out during spring 2020, when the first full lockdown was in effect, two-thirds of university students reported at least ‘much’ increased anxiety, and one-third reported increases in depressive feelings, while 2.59% reported an increase in suicidal thoughts [29]. Moreover, 12.43% reported major depression, and 13.46% reported severe distress. Risk factors included being female and having a history of self-injury and suicide attempts. In a different Greek study, Papastylianou and Zerva [30] reported that the levels of resilience and loneliness of emerging adult university students predicted life satisfaction (positively and negatively, respectively). During the first lockdown in Italy in the spring of 2020, Sanzò and colleagues [31] classified college students according to their levels of positive mental health as flourishing (25.96%) and as languishing (8.07%). Qualitative analysis of participant well-being and daily life experiences revealed that the domains of education and focus on inner life were associated with both challenges and satisfaction, while the necessary competences to face the pandemic successfully included resilience, coping strategies, hardiness, and optimism. During the lockdown in Mexico, López-Fuentes and Muñoz [32] empirically investigated resilience, self-efficacy, happiness, and perceived stress in young people, most of them university students. They suggested that youths with higher levels of resilience reported higher self-efficacy and lower perceived stress. 

Research evidence across the globe, including studies reported in the previous sections, are indicative of the importance attributed to the study of the various ways that the COVID-19 pandemic may impact resilience in emerging adult university students, and the role that pre-existing or current mental health problems may play in this relationship. In so doing, targeted interventions and programs may be developed to enhance youth resilience, well-being, and positive adaptation in times of crisis. 

The previous discussions set the framework for the present study. The following subsection delineates this study’s main aim as well as the research hypotheses and questions that guided it. 

### 1.3. Aim of the Study, Research Hypotheses, and Questions

This study seeks to study resilience in the face of perceived COVID-19-related negative impacts in emerging adult university students given their mental health status in the aftermath of the COVID-19 pandemic. Hence, the originality and contribution of this paper: it addresses a current point of international concern, as COVID-19 and its effects are still present and felt worldwide. It targets emerging adult students, a group that may be more vulnerable for developing mental health problems. Moreover, it is one of the few studies that directly measure the impact of COVID-19 on youths and its effects on resilience. Apart from measuring total pandemic-related impacts, the study also measures different types of impacts, i.e., financial, resource, and psychological, in connection with their relationships with resilience and mental health. It accomplishes this during a period seldom, if at all, studied in connection to the pandemic, namely its final couple of months. Further, it focuses on the relationship between resilience and mental health under different types and levels of pandemic-related impacts. Toward this end, it explores the potential moderating role of the impact of COVID-19 on the relation between youth resilience and mental health. In this manner, it attempts to uncover pathways to resilience in young university students under dire circumstances and crises, with a view to develop targeted psychosocial interventions and programs that promote positive youth development.

The research hypotheses guiding this study are delineated below.

**H1.**  *(Total) COVID-19 impacts will negatively predict resilience in emerging adult university students in the aftermath of the pandemic.*

**H2.**  *Different types of COVID-19 impacts, i.e., financial, resource, and psychological, will negatively predict resilience in emerging adult university students in the aftermath of the pandemic.*

The research questions in this study are:

**H3.**  *Does mental health moderate the relationship between (total) COVID-19 impacts and resilience in emerging adult university students in the aftermath of the pandemic?*

**H4.**  *Does mental health moderate the relationship between different types of COVID-19 impacts, i.e., financial, resource, and psychological, and resilience in emerging adult university students in the aftermath of the pandemic?*

The following sections describe (a) the materials and methods used in this study, including information about the study participants, the online questionnaire, the procedure, and the data analysis process; (b) the results, including descriptive statistics, as well as the relations between the impacts of the COVID-19 pandemic, resilience, and mental health in emerging adulthood; (c) a detailed discussion of the results, centering on their relevance to the extant literature, the confirmation or not of the research hypotheses and questions, and the implications for the theory and practice of resilience; and (d) the main conclusions drawn from the study.

## 2. Materials and Methods

This section describes various aspects of the methodology followed in this study. It details the socio-demographic characteristics of the sample, the questionnaire battery (which was answered online), the procedures followed, and the data analysis processes followed. 

### 2.1. Participants

In total, 205 female and male students at the University of Ioannina, in the northwest of Greece, participated in the study. Their age ranged between 18 and 29 years. Most were females, in accordance with the population of the departments and other studies. Table 1 reports sample demographics.

### 2.2. Materials

For the purposes of the study, a questionnaire battery was prepared for participants to complete online using Google Forms. It assessed (a) socio-demographic information, including age, gender, family socio-economic status, year of study, living at the place of study, type of accommodation, and satisfaction with living conditions; (b) impacts of the COVID-19 pandemic; (c) mental health; and (d) resilience. The scales used in the study can be downloaded using the hyperlink in Supplementary Materials.

1. The «Coronavirus Impacts Questionnaire» (Short. CIQ) [33] was used to assess the impact of the COVID-19 pandemic on aspects of daily life. This 6-item scale is scored on a Likert-type scale (1 = totally disagree and 7 = totally agree). Higher scores on the scale suggest higher impacts of the pandemic. The scale consists of three subscales, measuring financial, resource, and psychological (namely depression) impacts. Sample items include “The Coronovirus (COVID-19) has impacted me negatively from a financial point of view” and “I have had a hard time getting needed resources (food, toilet paper) due to the Coronavirus (COVID-19)”. The original scale is reported to have high internal reliability (financial subscale: α = 0.76, resource: α = 0.93, and psychological: α = 0.89). In the Greek sample, α = 0.71 for the total scale, α = 0.63 for the financial subscale, and α = 0.81 for both resource and psychological subscales.
2. The «GHQ-12» (General Health Questionnaire-12) [34] is a self-administered screening questionnaire for assessing common psychiatric disorders, namely depression, anxiety, and psychosomatic illness. The questionnaire comprises 12 items, scored on a 4-point Likert-type scale (1 = not at all to 4 = very often). Higher scores indicate higher levels of mental distress. Sample items include (In the past 4 weeks you have…) “lost much sleep over worry” and “Been able to face up to your problems”. In research with youths, Cronbach’s αs ranged from α = 0.83 to α = 0.88, and in this study α = 0.86.
3. The «Resilience Scale» (RS) [35,36], a 15-item scale was used to measure resilience as a dynamic process encompassing positive adaptation in the context of the COVID-19 pandemic. Items are scored using a 7-point Likert agree–disagree scale ranging from 1 = agree to 7 = disagree. All items are positively phrased so that a higher score indicates higher resilience. Sample items include “I usually manage one way or another” and “I can usually find something to laugh about”. The initial authors [35] report that the scale has concurrent validity with scales of morale, life satisfaction, and depression, and Neill and Dias [36] report Cronbach’s α = 0.91. Reliability in a Greek sample was α = 0.73, while Cronbach’s α in this study was α = 0.89.

### 2.3. Procedure

The Ethics and Deontology Committee of the University of Ioannina approved the study (N. 13260/13.3.2023). Students at various departments of the University of Ioannina were invited to participate in the online survey through announcements at their undergraduate and postgraduate lectures, with the consent of course tutors. Invitations to take part in the survey were additionally posted at the academic websites of the courses. Youths were also asked to invite other youths studying at the University of Ioannina aged 18–29 yrs to complete the survey. All participating students indicated their informed consent at the beginning of the online survey. The questionnaire was anonymous, and students could withdraw at any time. Participants completed the survey online at their own time. It took 12 min on average to complete. 

### 2.4. Data Analysis

A non-experimental, cross-sectional design was adopted in this study. An a priori power analysis was carried out using G*Power version 3.1.9.7 [37] to identify the minimum sample size necessary to test the study hypotheses. The results indicated that the required sample size to achieve over 90% power for detecting significant differences in a model of linear regression with 15 independent variables at a significance level of 5% was *n* = 200. The obtained sample size of *n* = 205 is adequate to test the study hypotheses. 

Preliminary analyses (means, standard deviations, partial correlations, Cronbach’s alphas) for all variables were performed first. Demographic differences in coronavirus impacts, mental health, and resilience were examined using independent samples *t*-tests and one-way ANOVAs. The potential role of GHQ as moderator in the relationship between COVID-19 impacts and resilience was tested using PROCESS v3.3 (model 1) [38]. With this method, significance is inferred if there is a significant interaction between the moderator and the independent variable, while conditional effects of the predictor variable at low, medium, and high levels of the moderator further elucidate the relationship between the various variables. All data analyses were performed using SPSS v22.0 (IBM, Armonk, NY, USA). 

In the next section, the results of the study are described in detail. Findings from the descriptive analyses are presented first to set the scene for results from further inferential analyses to be demonstrated subsequently. 

## 3. Results

### 3.1. Descriptive Statistics

Table 2 shows the means, standard deviations, Cronbach’s alpha internal reliability indices, and the partial correlations among total COVID-19 impacts, resilience, and mental health, including satisfaction with living conditions as a control variable (covariate). The latter was included in the correlations analysis since satisfaction with living arrangements was the sole demographic difference found: emerging adult university students who reported higher satisfaction with their living arrangements exhibited higher levels of resilience (F (2001, 3) = 3.2, *p* < 0.05). Therefore, satisfaction with living arrangements was included as covariate in all subsequent correlation, regression, and moderation analyses. 

All scales had adequate to high reliability, indicating that the sample could be representative of the population. The results also suggested that, toward the end of the COVID-19 pandemic, emerging adult students demonstrated moderate levels of total coronovirus impacts (CIQ) and mental health symptoms (GHQ) and moderate to high levels of resilience (RS). Moreover, coronovirus impacts were significantly and positively correlated with mental health but not with resilience. As expected, mental health was negatively correlated with resilience. 

In an effort to examine whether different types of COVID-19 impacts (i.e., financial, resource, and psychological) were differentially associated with mental health and resilience, and also among each other, partial correlations were carried out between all variables, including satisfaction with living conditions as covariate. The results, as shown in Table 3, indicated that students experienced moderate levels of financial, resource, and psychological impacts due to the COVID-19 pandemic. Analyses additionally revealed that COVID-19 financial impact was strongly and positively correlated with COVID-19 resource impact. In turn, resource impact was significantly and positively associated with the psychological impact of the pandemic. The latter was significantly and negatively correlated with resilience and positively correlated with mental health.

### 3.2. Impacts of the Pandemic, Resilience, and Mental Health in Emerging Adulthood

#### 3.2.1. Total Impacts of the Pandemic on Emerging Adult Resilience and Mental Health 

In order to understand the relations between total impacts of the COVID-19 pandemic, resilience, and mental health, a linear regression model was calculated. The model included resilience as dependent variable. Satisfaction with living arrangements was entered into the equation first, total COVID-19 impacts was added in the second step, and GHQ in the final step. The results indicated that COVID-19 impacts did not predict resilience; only satisfaction with living arrangements and GHQ predicted resilience (see Table 4 for details). 

#### 3.2.2. Financial, Resource, and Psychological Impacts of the Pandemic and Relations with Resilience and Mental Health in Emerging Adulthood

To further explore the relationships between the three types of pandemic-related impacts, mental health, and resilience, a linear regression analysis was estimated. With resilience as the dependent variable, satisfaction with living arrangements was entered into the regression equation first, followed by the three types of COVID-19 impacts at the second step, and mental health in the last step. The resultant model suggested that all steps were significant, with resource and psychological impacts significant at step 2 but only the former in step 3. Table 5 portrays the prediction of COVID-19 financial, resource, and psychological impacts as well as that of mental health on resilience for emerging adult university students. The results indicated that, at the last step of the regression equation, resilience was predicted by satisfaction with living arrangements, the impact relating to the resources available to emerging adult university students during the pandemic and mental health. 

### 3.3. Mental Health as a Moderator in the Relationship between COVID-19 Impacts and Resilience in Emerging Adulthood

To investigate whether mental health moderates the relationship between total, but also between the three types of COVID-19 impacts, and resilience, moderation analyses were carried out using PROCESS. In the first model, satisfaction with living arrangements was entered as control variable (i.e., covariate). The emergent models are shown in Table 6. The overall model was significant, *R* = 0.41, Δ*R*^2^ = 0.16, *MSE* = 0.76, *F* (4, 200) = 10.19, *p* < 0.001. The interaction between CIQ and GHQ additionally accounted for 17% of the variance (Δ*R*^2^ = 0.017, *F* (1, 200) = 3.98, *p* < 0.05). Subsequent simple slope analysis indicated that, under high levels of mental health symptoms, there is a significant positive relationship between COVID impacts and resilience (*b* = 0.17, 95% CI [0.02, 0.32], *t* = 2.26, *p* = 0.025). 

**Figure 1:**
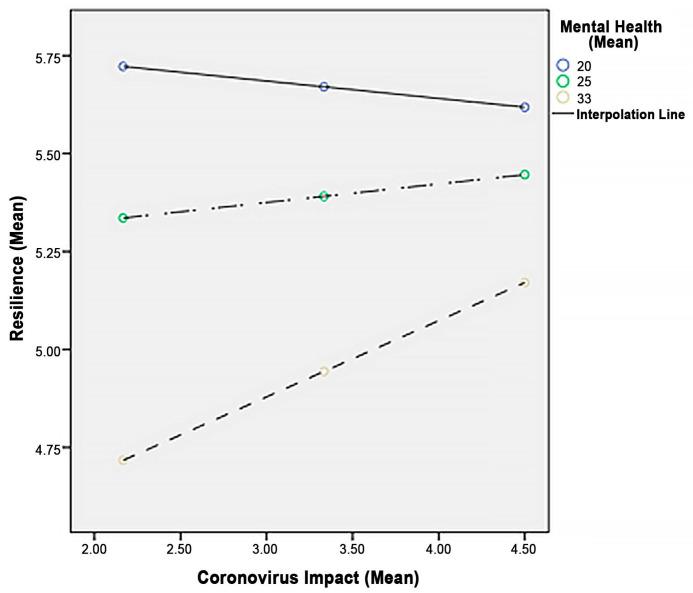
Mental Health (GHQ) as a Moderator between Total Coronovirus Impact (CIQ) and Resilience (RS).

**Figure 2:**
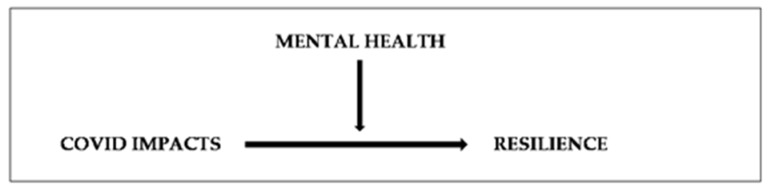
Diagram of the Moderating Conceptual Model of the Relationship between COVID Impacts and Resilience with Mental Health as a Moderator.

These results seem to indicate that the positive relationship between (total) COVID impacts and resilience is only observed in emerging adult university students with high levels of mental health symptoms and not in those youths with low or moderate levels of poor mental health. Figure 1 graphically represents the configuration of the variables. Figure 2 portrays the conceptual moderating model of the relationship between COVID impacts and resilience, with the mental health of emerging adult university students as a moderating variable. 

Analyses were also carried out to examine whether mental health moderated the relationship between financial, resource, and psychological impacts of the COVID-19 pandemic and resilience. As no evidence to that effect emerged, results are not reported here. 

The presentation of the results from this study having come to a conclusion, the following section discusses them in the light of previous research, as presented in the Introduction, but also in terms of theories that may begin to unravel the configuration of the variables under consideration. Cultural and family considerations are taken into account, in an attempt to decipher whether the various trajectories depicted in this research go some way toward explaining the impacts of the COVID-19 pandemic on the resilience and mental health of emerging adult students worldwide. 

## 4. Discussion

This study investigated the relationships between COVID-19 impacts and resilience of emerging adult university students as well as the potential moderating role of mental health symptoms in the aftermath of the pandemic. Overall, the results offered evidence on both accounts and are discussed in turn below. 

### 4.1. Discussing Demographics and Relations among Pandemic-Related Impacts, Mental Health, and Resilience in Emerging Adulthood

A number of socio-demographic characteristics of youths were expected to relate to the key study variables. As it happened, a single demographic variable was implicated in the relations between the key variables in this study. Satisfaction of young university students with their living situation was used as a control variable in partial correlation analyses, in regression analyses, and in moderation analyses. Moderate and high levels of satisfaction (73% to 80%) were repeatedly reported in research with studying youths in Greece [8,39]. Satisfaction was found to be positively correlated with indices of well-being, including resilience, flourishing, and positive perception [40]; it was found to be negatively correlated with sources of stress, and the character strengths of justice and temperance in emerging adult students [39]. It would appear that satisfaction with living arrangements assumes an important role during this period of life, complementing the developmental processes of individuation and forming romantic and social relations independently of the family system [2]. 

Moving on, significant partial correlations were revealed between perceived COVID-19 impacts, resilience, and mental health, when satisfaction of youths with their living arrangements were taken into account. For both total and the three types of COVID-19 impacts (financial, resource, and psychological), positive correlations were observed with mental health symptoms. Resilience was not correlated with total impacts but only with the psychological impact of COVID-19. This weak negative but still significant correlation (*r* = −0.13) was in the expected direction and, therefore, indicative of the pattern of relations between the detrimental effect of the psychological aspects of the pandemic on the resilience levels of emerging adult students. These tentative findings point to a crucial distinction between mental health symptoms and resilience. Both developmental and resilience science have long emphasized that absence of mental health problems does not signify presence of positive mental health and resilience [40,41,42,43]. 

In a similar manner, resilience and mental health symptoms were not directly associated with financial or resource impacts of the pandemic; rather, they were only associated with negative psychological impacts. To the degree that the latter were correlated with resource impacts, which were in turn correlated with financial impacts, a tentative link might be drawn between the aforementioned variables. A picture, then, seems to emerge whereby those affected more severely by financial impacts also faced problems obtaining adequate resources during the pandemic. The latter appeared to report higher psychological impacts due to the pandemic. These seemed to be expressed through their association with resilience (negative) and mental health problems (positive). These findings are suggestive of the centrality of the financial aspects and impacts of the pandemic and their adverse effects on mental health and resilience of studying youths [6]. This result resonates with findings in other recent studies. For instance, Argabright and colleagues [43] reported that the adverse financial effects of the pandemic significantly and negatively affected youth mental health, mostly through lost wages suffered by their families. As families represent a major force of financial support for children and youths, any loss of income adversely impact many aspects of the life of their offspring. Cui and Hong [21] reported on the pathways from family income loss in China during the pandemic to the mental health symptoms and resilience of young adults. They suggested that pandemic-related family income loss was associated with economic pressure, which was in turn related to negative parent–youth interactions, which led to anxiety and depressive symptoms in the latter. As a resilience proxy, self-control appeared to buffer the relation between family income loss and economic pressure, while respect for family, another resilience variable, weakened the link between economic pressure and negative parent–child interactions. Our own results, in outlining a potential route from the financial pandemic impacts to resilience and mental health may add to the growing literature in this domain but at the same time needs to be further explored with the use of longitudinal data and structural equation modeling techniques. Moreover, it should be noted that the measure of resilience used in this study was static in its nature and thus might not be able to capture the various adaptation processes at work with emerging adult students. More research is needed to identify other routes and processes leading to positive youth development. 

Moving along toward understanding processes involving resilience and mental health under the conditions of the latest pandemic, the next subsection discusses these issues with respect to emerging adult students. 

### 4.2. Predicting Resilience in Emerging Adult Students during the Pandemic 

To further elucidate the configuration between the key study variables, regression analyses were carried out. Perceived total COVID-19 impacts failed to predict resilience, contrary to the first study hypothesis (H1); however, satisfaction with living arrangements and mental health symptoms did. As the scale used to measure COVID-19 impacts (CIQ) is new, its psychometric properties are not extensively validated. In addition, CIQ is largely untested in relation to established mental health, well-being, and resilience measures. Therefore, this finding needs replication. 

With respect to different types of pandemic impacts, it was found that, apart from satisfaction with living arrangements and mental health symptoms, resilience was only predicted by resource impacts of COVID-19, thus partly confirming the second study hypothesis (H2). This finding differed from the one rendered by correlation analyses, which indicated that psychological, and not resource, impacts were associated with resilience. Nevertheless, as resource and psychological impacts were positively correlated, it is conceivable that they influence each other to confer resilience during a pandemic. This result is in line with international research of youth-led organizations carried out during the pandemic, which suggested that youth expressed the greatest concerns about mental health, disposable income, and employment impacts of the crisis [6]. Still, the relations between different types of COVID-19 impacts and indicators of mental health, well-being, and resilience need further exploration. 

In this light, the next subsection deals with the moderating role of mental health in the relationship between resilience and pandemic-related impacts in emerging adulthood. 

### 4.3. The Moderating Role of Mental Health

The COVID-19 pandemic clearly affected the mental health and resilience of emerging adult university students [5,6,9,10,24,25,26]. Findings seem to support a growing body of literature reporting a moderating, regulatory role for mental health symptoms with respect to resilience and well-being of youths in the aftermath of the COVID-19 pandemic [44,45]. Specifically, higher levels of mental health symptoms predicted lower levels of resilience, thus confirming the third study hypothesis (H3). Interestingly, although emerging adult students with low levels of symptomatology were more resilient, a decreasing trend on their resilience was observed when the level of the total pandemic impact augmented. This non-significant trend may be attributed to the generalized detrimental effect of the pandemic to all youths, independent of their levels of mental health symptoms. On the other hand, resilience increased as perceived total pandemic impacts and mental health symptoms increased, even though absolute levels of resilience remained low. Taken together, the above findings seem to suggest that absence of or reduced symptomatology may protect studying youths against vulnerability toward the end of the COVID-19 pandemic. 

The above findings may be approached from the viewpoint of the conservation of resources theory (COR) as proposed by Hobfall [46]. This theory suggests that broader life circumstances and resource-loss events interact to produce processes of resource conservation. Sometimes resource loss leads to further depletion of resources in a cyclical fashion. In such cases, individuals use resources available to them in order to adapt successfully. Successful adaptation generates new resources, which replenishes their resource pool and counterbalances the conditions that lead to resource loss, leading to gain spirals. In the case of this study, emerging adult students with pre-existing high levels of symptomatology and lower levels of resilience may be trapped in a loss spiral, where the negative impacts of the COVID-19 pandemic serve as external forces depleting their resources, which may well have been lower to begin with. On the other hand, youths with more resources to start with are considered most capable to restore or gain resources after severe adversity, such as the pandemic, even when they have high pre-existing symptomatology. Therefore, for young emerging adults it may be crucial to explore the resources available to them, if possible, prior to the onset of an adversity or crisis, in order to better understand any distinct resilience and adaptation processes they may exhibit. 

The study findings have different implications for youths facing low and moderate levels of mental health problems and different ones for those facing higher levels of symptomatology. For the former, it is possible that orchestrated efforts to reduce symptomatology, via interventions or counseling, for example, may directly enhance resilience in times of crisis. On the other hand, emerging adults with high levels of pre-existing or current symptomatology may well be used to handling mental health problems. Facing additional pandemic-related negative impacts may present a novel, higher-order challenge for them. This may mobilize them to resort to resources they already have access to and experience in using in order to meet the adversity facing them, according to the COR theory, as described above [46]. More targeted interventions to enhance resilience in these youths may be in order. These may seek to enrich their coping repertoire by developing additional, different types of skills to the ones they already possess and by obtaining access to resources they previously had not access to. More intensive exercises in using already existing and newly acquired skills may benefit these youths more. Also, allowing emerging adults a more active role in designing and training in handling specific pandemic-related stressors, as occurring in common everyday life contexts, may increase their ability to overcome crises more effectively. Further research is required to identify specific resources, individual, relational, organizational, community, or other, which may buffer the combined effects of symptomatology and augmented pandemic-related impacts. 

The final study hypothesis (H4) was not confirmed, since mental health failed to moderate the relationship between financial, resource, or psychological impacts of the pandemic and youth resilience. Perhaps cumulative pandemic-related stressors, especially when combined with mental health symptoms, impact resilience more decisively than any single type of impact alone. This would be in line with a corpus of evidence showing that the cumulative load of multiple stressors impacts mental health more severely than any one particular stressor [47]. Recently, in an investigation of the cumulative toll of pandemic stressors on the mental health consequences of pandemic-related stressors the authors found that those with three or more pandemic stressors exhibit more distress during the pandemic [48]. Mastery moderated stressors at higher levels of stress exposure, while social support and self-esteem became ineffective at the highest levels of pandemic stress. Further research should clarify whether any relationships exist between different types of COVID-19 impacts, mental health, and resilience, perhaps using a larger sample or a longitudinal design.

Having presented and interpreted the study results in relation to theory and other research findings from similar studies, other pertinent issues are discussed below. These include the study’s shortcomings but also its potential contribution to the theory and study of resilience in emerging adulthood under conditions of crisis. Lastly, recommendations for future research, practice, and public policy are presented in the following subsection.

### 4.4. Study Limitations, Contribution, Implications, and Recommendations for Future Research, Practice, and Public Policy

As in any research endeavor, this study has certain limitations, including sample representativeness. In particular, the sample was mostly drawn from a particular region in northwest Greece; moreover, there was uniformity to a large degree regarding the type of studies participants followed: they largely studied pedagogics and psychology. Therefore, the results may be limited to the above groups. As is common in research in the social sciences, there was a gender imbalance in this study—nonetheless, the rate of around 85% for females found here is comparable to that found in similar studies. In any case, the results need to be cautiously interpreted, since they may be particular to young women. Young women who live at home may be more protected against adversity, as some studies report [48]. Therefore, they may exhibit higher resilience levels. More gender balanced studies are needed for results to be applicable to young men as well. In terms of participant age, mostly younger emerging adults aged 18–21 years took part in this study (83%). In a similar study, Serrano Sarmiento and colleagues [49] found that youths aged 17–20 years displayed high and solid resilience to a higher degree than those in middle emerging adulthood. Therefore, caution is required in the interpretation of the study results. In addition, to the degree that youths in middle and late emerging adulthood were under-represented, the generalization of results to these groups is limited. Moreover, the sample consisted of studying youths. There is currently a need to study other groups of emerging adults, including NEETs (not in education, employment, or training), young people in part-time employment, or those occupied in family businesses. 

Another study limitation needs to be addressed at this point. Satisfaction with living conditions, a variable clearly implicated in the relationships among the variables in this study, is closely linked to whether students resided in their family home with their family or in alternative accommodation. A fifth of the youths who lived in their place of study resided in the family home. This may have repercussions for their psychosocial development, resilience, and mental health under crises, especially as far as autonomy and individuation, both key needs and processes for this age group [2,50]. In particular, young emerging adults tend to seek financial, social, and emotional independence from their parents, which may be easier to achieve when they live away from home. Going away to study represents a major transition for young people, providing an excellent opportunity to explore their identity more fully [51]. It is worth exploring whether living on their own for the first time tends to increase or decrease their resilience. Research evidence is mixed, as some studies suggest that the family in Greece and abroad increases resilience [30,52]. Yet, other studies indicate that living on their own may decrease resilience in young people, especially during the confinement due to COVID-19, when resilience was higher among university students living alone or with other people, rather than with their parents [52]. Family relationships and dynamics may be negatively impacted during adversity, as the literature on the impact of COVID-19 on family and offspring resilience, well-being, and overall adaptation suggests [48]. The extent to which severe crises will impact families and young people largely depends on other factors and processes in their lives, such as mechanisms related to building and maintaining support networks [53,54] and to optimizing family belief systems to better understand pandemic-related events [53,55,56]. Future studies may wish to look more exhaustively into these issues. 

Other study limitations center on measurement. The CIQ is a new scale, not extensively tried in psychosocial research, and would benefit from validation against other pandemic-related scales. Furthermore, all scales used in the study relied on self-report and are, therefore, subject to various biases. A more integrated approach, involving the use of other methods, such as observation and neurophysiological measures, would improve understanding of the relations between the study variables. Multiple respondents would also provide richer perspectives of the phenomena under study. A mixed-methods approach might also allow for fuller comprehension of the relations between variables. A longitudinal research design might enable the inference regarding causality or direction of the results. These shortcomings should be considered during interpretation and application of the study findings in counseling or educational settings.

The above limitations notwithstanding, this original study sought to contribute to the expanding literature on the effects of the COVID-19 pandemic on a number of counts. It attempted to answer scientific calls to explore issues around the COVID-19 pandemic and its impact on mental health and resilience [5,6]. It focused on emerging adults, a group who many researchers consider potentially more vulnerable to external crises: apart from the inherent instability in many life domains that youths need to face and the life changing decisions they are called to make, external severe crises, such as the latest pandemic, may amount to cumulative stress, with potentially severe negative implications for their mental health, well-being, and resilience [48]. In addition, the study concentrated on university students as the pandemic-related educational disruptions brought about by government-imposed measures to contain the spread of the virus, such as lockdowns, social isolation, and loss of family income, arguably affect their education and career prospects as well as social relations [24,26,27]. One of the central features of this study pertained to its use of a pandemic-specific questionnaire instead of inferring the impact of the pandemic on participants. Lastly, the study was carried out toward the end of the pandemic, thus possibly allowing for a unique view at an understudied period of the pandemic so far. 

The central question of the study pertained to the possible moderating role of mental health symptoms in the relationship between total and specific impacts of the COVID-19 pandemic, including financial, resource, and psychological impacts, with resilience. The resultant findings tentatively allow for (a) description of relations between the study variables but perhaps more importantly suggest (b) a regulatory role for mental health symptoms. Describing two distinct pathways to youth resilience under the pandemic, one for those experiencing low and moderate levels of mental health symptoms and a different one for those suffering high levels of symptomatology, may be useful. It may suggest that research efforts could be usefully expended toward uncovering different moderators and diverse configurations of psychosocial factors, with a view to increase resilience during unexpected, severe, and impactful future crises and health pandemics. 

The study findings may prove important at a research level, and they also may hold implications for the practice of resilience during severe crises. They may facilitate and direct the design and implementation of interventions and programs, be they educational, clinical, or counseling in nature in order to enhance resilience in emerging adulthood, especially for studying youths. Targeted interventions for each group can focus on strengthening different aspects of individual or relational skills and resources of young people and also on offering youths tailored support in facing any pre-existing mental health problems or any cumulative effects stemming from any pandemic-related stressors they may additionally be called to handle. Focusing on a different aspect of resilience interventions during crises, it may be useful to capitalize on youth skills that were acquired or honed during the COVID-19 pandemic. As barriers to in-person interventions took place, virtual (online) interventions surfaced at an unprecedented scale, be they self-paced, peer-led, or group-based [57]. Younger populations are uniquely situated to take advantage of and move such initiatives forward, as they are more at ease with the use of technology tools and platforms than older generations. Their current mobilization in alleviating the direr effects of the pandemic through, for instance, digital tools and data may facilitate building up resilience at an individual and societal level against future crises and disasters [6].

This paper focused on the individual: the effects of the COVID-19 pandemic were examined at an individual level, as were mental health symptomatology and resilience. Crises and pandemics, however, have by definition wider scopes and repercussions, including the family, societal institutions, governments, and international organizations. Therefore, no discussion of pandemic-related stressors and outcomes can be complete without reference to the public, health, and employment policies and concerns [6]. Young people may act as catalysts in strengthening the resilience of institutions, governments, and societies to safeguard against future crises, ensuring at the same time the resilience and well-being of youths now and in the future. According to a 2020 OECD [6] report, “already prior to the COVID-19 pandemic, young people have been at the forefront of calls for a longer-term perspective in policy making and in building more inclusive and sustainable societies, for instance through a transition to greener economies”. Harnessing this youthful dynamic in an effort to inform policy in different societal fields may be necessary to build societal resilience. For example, in order to update national youth strategies to mitigate the effects of crises, young stakeholders need to be consulted in order to translate political will into actionable programs [6]. Additionally, national statistical offices and research institutes could offer vital aid in assembling disaggregated information on the impact of the crisis in youths by way of tracking inequalities and informing decision making [ibid]. 

As a final note, the questions that guided this study and the findings that it generated fall directly in the scope of a newly carved field of enquiry. This field deals with the impacts of severe crises, disasters, or health pandemics on the mental health, well-being, and resilience of individuals, communities, institutions, and societies [5,6]. See Leontopoulou and Delle Fave [58] for a recent collection of papers in this area focusing on emerging adulthood). It seeks to understand the relations among stressors, resources, and adaptive outcomes in various populations. The field is informed by developmental science and mental health, resilience studies, educational and social psychology, and counseling, to name but a few. These and many more scientific fields stand to benefit from the progress made at a research and applied level. Furthermore, the concerned disciplines are now better equipped to address relevant questions using international, cross-sectional, or, preferably, longitudinal, data as well as quantitative, qualitative or mixed methodologies and complex analytic techniques and tools. 

Having discussed the research findings, the main conclusions of the study are presented below, with a view to render the main issues addressed in this study more concrete and to suggest ways that the findings may inform different areas of research enquiry. 

## 5. Conclusions

In conclusion, this study explored the relationship between COVID-19 impacts and resilience in emerging adult university students at the aftermath of the pandemic. It made tentative progress toward mapping the relations between total and also financial, resource, and psychological pandemic-related impacts with resilience, under the influence of mental health symptoms. In addition, it suggested that mental health symptoms may moderate this relationship. Interpreting this finding, two distinct pathways seemed to emerge. The first suggested that studying youths who experience low or moderate levels of symptomatology are more resilient, independently of the level of pandemic-related stressors they may face. For them, interventions need to focus on maintaining resilience, by introducing skills and enhancing already existing individual and relational resources they may possess. The second pathway indicated that emerging adult university students experiencing higher levels of mental health symptoms, in tandem with high levels of pandemic-related impacts, exhibit increasingly higher resilience levels. This seemingly unorthodox finding may be indicative of a different type of mechanism at work in this situation. These youths may be better equipped to handle severe stress and adversity, thanks to skills and resources they possess and are experienced in using. This line of reasoning may lead to a recommendation for more in-depth examination of the specific types and levels of resilience skills required to deal with deep-rooted or cumulative stressors under conditions of severe crisis, such as health pandemics. 

This study falls into the scope of a new line of scientific inquiry, that of severe crises, disasters, and pandemics, the relations and dynamics that take place within their contexts, and the psychological and social responses they evoke [5,6,58]. In essence, the emerging field is informed by a number of research disciplines, including, but not limited to, developmental science and mental health, resilience studies, educational research, artificial intelligence studies, and public health and employment policies. It is hoped that as research and practice expands within this field, individual, family and institutional resilience will increase to protect against current and future public health emergencies and other crises and to help individuals and societies flourish.

## Figures and Tables

**Table 1:** Demographic characteristic of study participants.

Demographic Variables		Emerging Adults University Students (*N* = 205)
Age	18–21 yrs	170 (82.9%)
	22–25 yrs	29 (14.1%)
	26–29 yrs	6 (2.9%)
Gender	Female	174 (84.9%)
	Male	29 (14.1%)
	Other	2 (1%)
Family SES	Lower	22 (10.7%)
	Medium	157 (76.6%)
	Higher	9 (4.4%)
	N/A	17 (8.3%)
Year of study	1st yr	87 (42.4%)
	2nd yr	53 (25.9%)
	3rd yr	34 (16.6%)
	4th yr	23 (11.2%)
	>4th yr	8 (3.9%)
Live in place of study	Yes	183 (89.3%)
	No	22 (10.7%)
Accommodation, if Yes	Family	43 (21%)
	Alone	89 (43.4%)
	Roommates	21 (10.2%)
	Relatives	10 (4.9%)
	Hall of Residence	15 (7.3%)
	Partner	6 (2.9%)
Accommodation, if No	Family	20 (9.8%)
	Alone	13 (6.3%)
	Relatives	4 (2%)
	Hall of Residence	15 (7.3%)
	Partner	2 (2.9%)
Satisfaction with living conditions	Not at all	3 (1.5%)
	A little	14 (6.8%)
	Moderate	67 (32.7%)
	A lot	121 (59%)

**Table 2:** Means, standard deviations, Cronbach’s alphas, and partial correlations between perceived COVID-19 impact, mental health, and resilience, with satisfaction with living arrangements as covariate.

Scales ^1^	M (SD)	Cronbach’s Alpha	CIQ	GHQ
CIQ	3.36 (1.18)	0.71	-	
GHQ	26.3 (6.36)	0.86	0.21 **	-
RS	5.35 (.94)	0.89	0.00	0.33 ***
[Satisfaction with living arrangements]	3.36 (1.18)	-		

^1^ CIQ: Coronavirus Impacts Questionnaire. GHQ: General Health Questionnaire-12. RS: Resilience Scale. ** *p* < 0.01, *** *p* < 0.001.

**Table 3:** Means, standard deviations, Cronbach’s alphas, and partial correlations between types of perceived COVID-19 impact, mental health, and resilience, with satisfaction with living arrangements as covariate.

Scales ^1^	M (SD)	Cronbach’s Alpha	RS	GHQ	CIQ Financial	CIQ Resource
RS	5.35 (0.94)	0.86	-			
GHQ	26.3 (6.36)	0.89	−0.35 ***	-		
CIQ Financial	3.22 (1.48)	0.63	0.04	0.00	-	
CIQ Resource	3.21 (1.65)	0.81	0.11	0.08	0.48 ***	-
CIQ Psychological	3.63 (1.8)	0.81	−0.13 *	0.33 ***	0.12	0.22 ***
[Satisfaction with living arrangements]	3.49 (0.69)	-				

^1^ RS: Resilience Scale. GHQ: General Health Questionnaire-12. CIQ: Coronavirus Impacts Questionnaire: Financial, Resource, and Psychological Impacts subscales. * *p* < 0.05, *** *p* < 0.001.

**Table 4:** Linear Model of Predictors of Resilience: Satisfaction with Living Conditions, Total Perceived COVID-19 Impacts, and Mental Health.

Independent Variables	*B*	*T*	*Sig T*	*R* ^2^	Δ*R*^2^	Δ*F*
Satisfaction with living arrangements	0.15	2.36	0.01 **	0.04	0.04	8.36 **
CIQ	0.07	1.16	0.24	0.03	0.00	0.95
GHQ	−0.34	−5.18	0.00 ***	0.14	0.11	26.86 ***

** *p* < 0.01 *** *p* < 0.001.

**Table 5:** Linear Model of Predictors of Resilience: Satisfaction with Living Conditions, Types of Perceived COVID-19 Impacts, and Mental Health.

Independent Variables	*B*	*T*	*Sig T*	*R* ^2^	Δ*R*^2^	Δ*F*
Satisfaction with living arrangements	0.14	2.23	0.026 *	0.03	0.04	8.36 **
CIQ Financial	−0.02	−0.35	0.724	0.06	0.04	2.97 *
CIQ Resource	0.17	2.26	0.025 *			
CIQ Psychological	−0.06	−0.9	0.369			
GHQ	−0.032	−4.68	0.000 ***	0.15	0.09	21.94 ***

* *p* < 0.05, ** *p* < 0.01, *** *p* < 0.001.

**Table 6:** Linear Model of Predictors of Resilience: Total Perceived COVID-19 Impact and Mental Health with Satisfaction with Living Conditions as Covariate.

Variable	*B*	*SE*	*t*	*p*	95% CI
CIQ	−0.38	0.23	−1.67	0.09	[−0.84, 0.69]
GHQ	−0.11	0.03	−3.56	0.00	[−0.17, −0.49]
CIQ × GHQ	0.01	0.009	1.99	0.04	[0.00, 0.34]
Satisfaction with living arrangements	0.20	0.09	2.25	0.02	[0.02, 0.37]

× = Interaction between CIQ and GHQ.

## Data Availability

The data are not publicly available due to restrictions on privacy.

## Supplementary Materials

The following supporting information can be downloaded at: https://www.mdpi.com/article/10.3390/ijerph20206911/s1. The scales used in the study can be accessed using the hyperlink.
