# Supplementary material for: Aftermath of the COVID-19 Pandemic: Resilience and Mental Health of Emerging Adult University Students
Source: Int J Environ Res Public Health. 2023 Oct 11;20(20):6911. doi: 10.3390/ijerph20206911 (PMC10606035; doi:10.3390/ijerph20206911)
Supplement: Supplementary file 1 [file ijerph-20-06911-s001.zip › ijerph-2564623-supplementary.pdf]

*Supplementary Materials*

# Aftermath of the COVID-19 Pandemic: Resilience and Mental Health of Emerging Adults University Students

**Sophie Leontopoulou**

Department of Primary Education, University of Ioannina, 45110 Ioannina, Greece; sleon@uoi.gr

The scales used in the study are presented below.

**1. Coronavirus Impacts Questionnaire (Short version)**

1. The coronavirus (COVID-19) has negatively affected me from a financial point of view.
   1 2 3 4 5 6 7
   Totally Disagree — Neither Agree, nor Disagree — Totally Agree
2. I have lost income related to my employment due to the coronavirus (COVID-19).
   1 2 3 4 5 6 7
   Totally Disagree — Neither Agree, nor Disagree — Totally Agree
3. I have had difficulties finding necessary supplies (food, toilet paper) due to the coronavirus (COVID-19).
   1 2 3 4 5 6 7
   Totally Disagree — Neither Agree, nor Disagree — Totally Agree
4. It was difficult for me to find things I need due to the coronavirus (COVID-19).
   1 2 3 4 5 6 7
   Totally Disagree — Neither Agree, nor Disagree — Totally Agree
5. I have been depressed due to the coronavirus (COVID-19).
   1 2 3 4 5 6 7
   Totally Disagree — Neither Agree, nor Disagree — Totally Agree
6. The coronavirus (COVID-19) pandemic has negatively affected my mental health.
   1 2 3 4 5 6 7
   Totally Disagree — Neither Agree, nor Disagree — Totally Agree

**2. Short General Health Questionnaire (GHQ 12)**

**During the last 4 weeks, have you:**

1. Been able to concentrate on what you're doing?
1 = Better than usual 2 = Same as usual 3 = Less than usual 4 = Much less than usual
2. Lost much sleep over worry?
1 = Not at all 2 = No more than usual 3 = Rather more than usual 4 = Much more than usual
3. Felt you were playing a useful part in things?
1 = More so than usual 2 = Same as usual 3 = Less useful than usual 4 = Much less useful
4. Felt capable of making decisions about things?
1 = More so than usual 2 = Same as usual 3 = Less so than usual 4 = Much less capable
5. Felt constantly under strain?
1 = Not at all 2 = No more than usual 3 = Rather more than usual 4 = Much more than usual
6. Felt you couldn't overcome your difficulties?
1 = Not at all 2 = No more than usual 3 = Rather more than usual 4 = Much more than usual
7. Been able to enjoy your normal day-to-day activities?
1 = More so than usual 2 = Same as usual 3 = Less so than usual 4 = Much less than usual
8. Been able to face up to your problems?
1 = More so than usual 2 = Same as usual 3 = Less so than usual 4 = Much less able
9. Been feeling unhappy and depressed?
1 = Not at all 2 = No more than usual 3 = Rather more than usual 4 = Much more than usual
10. Been losing confidence in yourself?
1 = Not at all 2 = No more than usual 3 = Rather more than usual 4 = Much more than usual
11. Been thinking of yourself as a worthless person?
1 = Not at all 2 = No more than usual 3 = Rather more than usual 4 = Much more than usual
12. Been feeling reasonably happy, all things considered?
1 = More so than usual 2 = About same as usual 3 = Less so than usual 4 = Much less than usual

**3. Resilience Scale**

1. When I make plans I follow through with them.
   1 2 3 4 5 6 7
   Totally Disagree — Neither Agree, nor Disagree — Totally Agree
2. I usually manage one way or another.
   1 2 3 4 5 6 7
   Totally Disagree — Neither Agree, nor Disagree — Totally Agree
3. I feel proud that I have accomplished things in my life.
   1 2 3 4 5 6 7
   Totally Disagree — Neither Agree, nor Disagree — Totally Agree
4. I usually take things in my stride.
   1 2 3 4 5 6 7
   Totally Disagree — Neither Agree, nor Disagree — Totally Agree
5. I am friends with myself.
   1 2 3 4 5 6 7
   Totally Disagree — Neither Agree, nor Disagree — Totally Agree

6. I feel that I can handle many things at a time.

| 1 | 2 | 3 | 4 | 5 | 6 | 7 |
|---|---|---|---|---|---|---|
| Totally Disagree | | | Neither Agree, nor Disagree | | | Totally Agree |

7. I am determined.

| 1 | 2 | 3 | 4 | 5 | 6 | 7 |
|---|---|---|---|---|---|---|
| Totally Disagree | | | Neither Agree, nor Disagree | | | Totally Agree |

8. I have self-discipline.

| 1 | 2 | 3 | 4 | 5 | 6 | 7 |
|---|---|---|---|---|---|---|
| Totally Disagree | | | Neither Agree, nor Disagree | | | Totally Agree |

9. I keep interested in things.

| 1 | 2 | 3 | 4 | 5 | 6 | 7 |
|---|---|---|---|---|---|---|
| Totally Disagree | | | Neither Agree, nor Disagree | | | Totally Agree |

10. I can usually find something to laugh about.

| 1 | 2 | 3 | 4 | 5 | 6 | 7 |
|---|---|---|---|---|---|---|
| Totally Disagree | | | Neither Agree, nor Disagree | | | Totally Agree |

11. My belief in myself gets me through hard times.

| 1 | 2 | 3 | 4 | 5 | 6 | 7 |
|---|---|---|---|---|---|---|
| Totally Disagree | | | Neither Agree, nor Disagree | | | Totally Agree |

12. I can usually look at a situation in a number of ways.

| 1 | 2 | 3 | 4 | 5 | 6 | 7 |
|---|---|---|---|---|---|---|
| Totally Disagree | | | Neither Agree, nor Disagree | | | Totally Agree |

13. My life has meaning.

| 1 | 2 | 3 | 4 | 5 | 6 | 7 |
|---|---|---|---|---|---|---|
| Totally Disagree | | | Neither Agree, nor Disagree | | | Totally Agree |

14. When I am in a difficult situation, I can usually find my way out of it.

| 1 | 2 | 3 | 4 | 5 | 6 | 7 |
|---|---|---|---|---|---|---|
| Totally Disagree | | | Neither Agree, nor Disagree | | | Totally Agree |

15. I have enough energy to do what I have to do.

| 1 | 2 | 3 | 4 | 5 | 6 | 7 |
|---|---|---|---|---|---|---|
| Totally Disagree | | | Neither Agree, nor Disagree | | | Totally Agree |
